# Craniofacial Microsomia: Goldenhar Syndrome in Association with Bilateral Congenital Cataract

**DOI:** 10.1155/2015/435967

**Published:** 2015-10-08

**Authors:** U. D. Shrestha, S. Adhikari

**Affiliations:** ^1^National Academy of Medical Sciences, Kathmandu, Nepal; ^2^Tilganga Institute of Ophthalmology, P.O. Box 561, Gaushala, Kathmandu, Nepal

## Abstract

Craniofacial microsomia (CFM) includes a spectrum of malformations primarily involving structures derived from the first and second branchial arches. Patients with hemifacial microsomia and epibulbar dermoids are said to have Goldenhar syndrome (GHS). Four-month-old boy with whitish pupillary reflex presented with the features of GHS in pediatric ophthalmology clinic. The child had ocular and auricular manifestations. There were no vertebral anomalies, but he had bilateral congenital cataract. The peculiarity of this case is the presence of the bilateral total congenital cataract, in association with CFM. There is absence of epibulbar dermoid or lipodermoid in the eyes, although the child had features of GHS. In addition to it, anesthetic intubation was smooth in this case. Any case diagnosed with CFM and/or GHS needs treatment through multidisciplinary approach, consultation in ophthalmology department is one of them.

## 1. Introduction

CFM includes a spectrum of malformations primarily involving structures derived from the first and second branchial arches. Characteristic findings include facial asymmetry resulting from maxillary and/or mandibular hypoplasia; preauricular or facial tags; ear malformations that can include microtia (hypoplasia of the external ear), anotia (absence of the external ear), or aural atresia (absence of the external ear canal); and hearing loss and ocular abnormalities. Hemifacial microsomia often refers to craniofacial microsomia with maxillary or mandibular hypoplasia. People with hemifacial microsomia and epibulbar dermoids may be said to have GHS or oculoauricular dysplasia. The pathognomonic triad of GHS includes CFM, dermolipoma, and vertebra skeletal anomalies [1].

## 2. Case Presentation

### 2.1. Chief Complaint

Four-month-old baby was referred with the chief complaint of white pupillary reflex in both the eyes on 14 July, 2013. The antenatal history was uneventful. The patient was born at 36 weeks of gestation and was delivered by normal spontaneous vaginal delivery (NSVD). The child did not have major illness after the delivery. He was not admitted in intensive care unit (ICU). There was no family history of facial syndromes or ocular conditions.

### 2.2. Ocular Examination

The child followed the light with both eyes (OU). Eyelids were normal OU.

**Figure 1:**
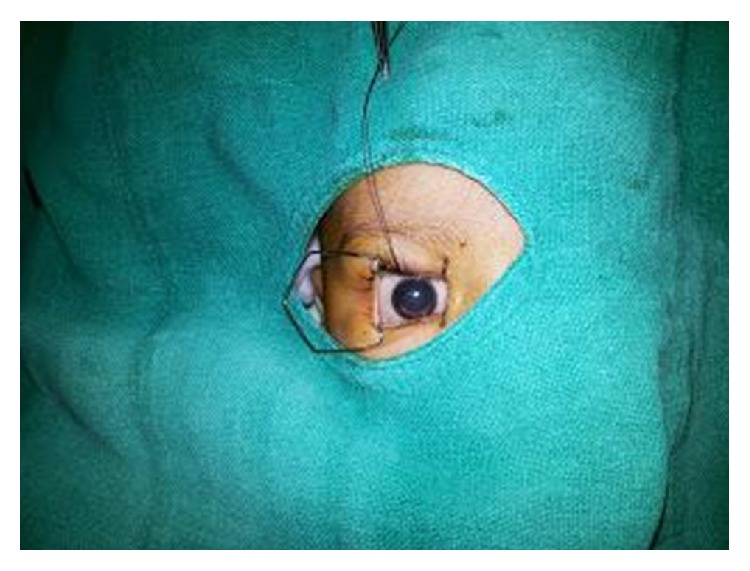
Child with cataract in the right eye.

**Figure 2:**
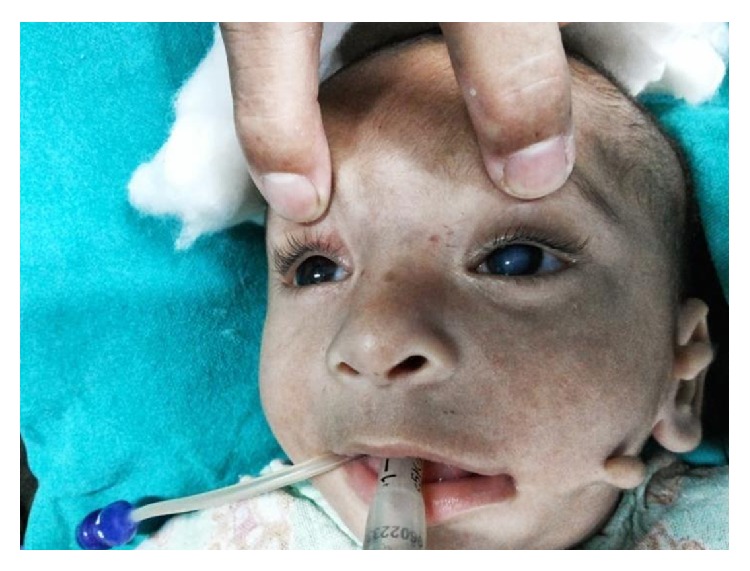
Child with cataract in the left eye and preauricular tag.

**Figure 3:**
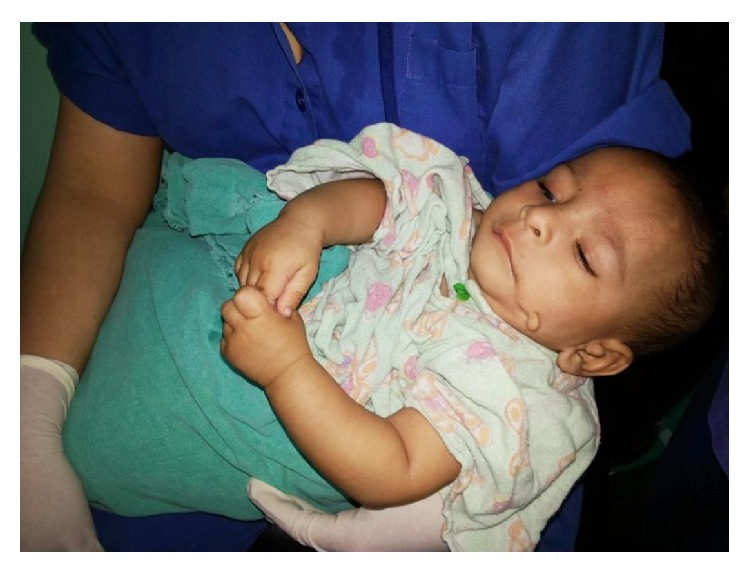
Child with preauricular tag and the skin tag in the cheek.

Extraocular motility was full in all cardinal positions of gaze. Nystagmus was present. Anterior segment examination revealed normal conjunctiva and cornea. Pupils were round, regular, and reacting, OU. On the lens, total cataract was seen, OU (Figures 1 and 2). Fundus was not visible. Hence ultrasound was done. There were no echo dense opacities, OU. On facial examination the child had facial asymmetry, presence of skin tags on left cheek. Preauricular tags were present on both sides (Figures 2 and 3). He had high arched palate.

For both the eyes, lens aspiration with anterior vitrectomy was planned under general anesthesia, as the child was only four months old. Left eye was operated on 9 August, 2013. Right eye was operated on 30 August, 2013. Difficult intubation is expected in GHS patients because of facial and oral abnormalities especially mandibular hypoplasia and limitation of neck movement resulting from vertebral anomalies. In this case the anesthetic intubation was smooth. There was no difficulty in airway management [2]. No single airway test can provide a high index of sensitivity and specificity for prediction of difficult airway in patients of GHS. The airway and anesthetic management of GHS patients depend on the type, extent, and severity of craniofacial-vertebral anomalies, associated cardiovascular problems, and nature of surgery.

Postoperatively, retinoscopy and refraction were done in four weeks after the cataract surgery.

He was prescribed the aphakic glass of +16.00 D. On 3-month follow-up, the child had alternate fixation with the glasses.

## 3. Discussion

CFM describes the spectrum of abnormalities that primarily affect the cranial and facial development before birth. CFM patients have microsomia, facial asymmetry between the two sides. In about two-thirds of cases, facial asymmetry is bilateral. The facial characteristics in CFM typically include unilateral maxillary or mandibular hypoplasia. It results in dental problem and breathing, feeding, and speech difficulty.

GHS is also known as oculoauricular-vertebral spectrum or variant of hemifacial microsomia. It is a developmental anomaly of maxillofacial skeleton, apparent at birth. It results in macrostomia and lateral facial clefts. GHS is dysmorphogenesis which occurs due to aberrant development of the first and second branchial arches [3]. It has prevalence from 1 to 9/100000 live births and incidence rate is 1 per 25000–45000 births, with a male to female ratio of 3 : 2.

It is mostly unilateral (70–80%). Anomalies in patients with this syndrome include preauricular skin tags (90%), hemifacial microsomia (77%), vertebral anomalies of different size and shape (70%), microtia (52%), CNS malformations (47%), cardiac malformations (39%), epibulbar dermoid cyst (39%), unilateral maxillary hypoplasia, and genitourinary anomalies [4, 5]. Oral cavity malformations include high arch or cleft palate and tongue abnormalities. This patient had the high arched palate [6, 7].

Other ocular anomalies include microphthalmos, microcornea, and anophthalmos. Deficient neural crest migration into the developing eye can lead to coloboma which can be present in eyelid, iris, and choroid [8]. Corneal hypoesthesia, extraocular motility disorders like strabismus, blepharoptosis, anomalous lacrimal drainage system, polar cataract, and retinal and optic nerve anomalies may be present.

Microtia can result from aberrant migration of neural crest cells into the first and second branchial arches during early embryonic development.

GHS is known for its classical triad of epibulbar dermoids or lipodermoids, auricular appendages, and pretragal fistulas [8]. However, this child did not have any epibulbar dermoid. According to Baum and Feingold, 24% of cases do not have any epibulbar dermoids, 23% have bilateral dermoid, and 53% have unilateral epibulbar dermoid [9]. Polydactyl is one of the unusual associations in GHS [10].

This is the case report to describe GHS in association with bilateral congenital total cataract. Congenital cataract is the opacification of the lens and its capsule. The aetiology of the cataract is: in one-third cases-idiopathic, in one-third cases- maternal infections and the rest of one-third casesgenetic and hereditary.

Developmental genes PAX 6 and PITX3 affect the lens development [11]. Any problem during embryonic lens development leads to congenital cataract formation.

The most common aetiologies of all pediatric cataract include intrauterine infections, metabolic disorders, and genetically transmitted syndromes [12]. However, CFM most frequently occurs as a simplex case with unknown etiology. It is unclear what genes are involved in CFM. It is not well understood why certain disruptions to development affect the first and second pharyngeal arches in particular.

Although the etiology of this disease is not fully understood, autosomal recessive or dominant inheritance is possible. The majority of patients with hemifacial microsomia including GHS are sporadic cases [13].

CFM and GHS are diagnosed clinically. They are the diagnoses of exclusion. Genetic study is important nowadays. However, till now no definite genetic test has been described to diagnose CFM and GHS yet [14].

## 4. Conclusion

The peculiarity of this case is the presence of the bilateral total congenital cataract, in association with CFM. There is absence of epibulbar dermoid or lipodermoid in the eyes, although the child had features of GHS. In addition to it, anesthetic intubation was smooth in this case. Congenital cataract should be considered as one associations of the GHS. Any case diagnosed with CFM and/or GHS needs treatment through multidisciplinary approach, consultation in ophthalmology department is one of them.
